# Validation of an Enzyme‐Linked Immunosorbent Assay for Measuring Leptin, a Key Metabolic Hormone, in Dried Blood Spot Samples

**DOI:** 10.1002/ajhb.70172

**Published:** 2025-12-04

**Authors:** Elizabeth Y. Kim, Luna S. Orozco, Emma G. Shoemaker, Jeffrey Gassen, Tomasz J. Nowak, Sally P. Weaver, Erich J. Baker, Michael P. Muehlenbein, Samuel S. Urlacher

**Affiliations:** ^1^ Department of Biology Baylor University Waco Texas USA; ^2^ Department of Anthropology Baylor University Waco Texas USA; ^3^ Department of Psychiatry University of California Los Angeles Los Angeles California USA; ^4^ Waco Family Medicine Waco Texas USA; ^5^ Department of Computer Science Belmont University Nashville Tennessee USA

**Keywords:** anthropology, biomarkers, dried blood spots, human biology, leptin, non‐invasive

## Abstract

**Objectives:**

Leptin is an established biomarker of appetite regulation and energy status. Problematically, heavy reliance on invasive venipuncture sampling has limited leptin research with diverse human populations and groups such as children. Key questions remain about leptin's evolution and biological roles across the full range of humans. Here, we present and validate a new minimally invasive approach for measuring leptin in finger‐prick dried blood spots (DBS) using a commercial ELISA kit.

**Methods:**

The Human Leptin Quantikine QuicKit ELISA (R&D Systems, QK398) was validated using matched serum and DBS samples from 40 adults. Passing–Bablok regression assessed the relationship between leptin_DBS_ and leptin_serum_. Dilutional linearity, reliability, spike‐and‐recovery, limit of detection, and stability tests evaluated assay performance and potential DBS matrix interference.

**Results:**

Leptin was reliably measured in all DBS samples (average = 312 pg/mL), with DBS intra‐ and inter‐assay CVs of 3.3% and 2.0%, respectively. Matched leptin_DBS_ and leptin_serum_ measurements showed excellent agreement (Pearson's *R* = 0.97), with no apparent bias (Bland‐Altman bias = 4.7). Leptin measurement in DBS was stable for at least 72 h at 26.2°C and 37°C and showed no degradation across eight freeze–thaw cycles (*p* > 0.05).

**Conclusions:**

Leptin can be reliably and stably measured in minimally invasive DBS samples, expanding research on energetics and appetite regulation across a wider range of human groups and settings.

## 1 Introduction

Leptin, a 16‐kilodalton polypeptide hormone encoded by the *ob* gene, plays a central role in appetite regulation and energy balance. Secreted by adipocytes in proportion to fat mass and able to cross the blood–brain barrier, leptin supports homeostatic control of energy reserves via inhibitory effects on appetite (Perakakis et al. 2021).

As a biomarker in clinical and population research, leptin illuminates complex mechanisms underlying body weight regulation and metabolism. Leptin has been studied extensively in the context of obesity and related metabolic disorders such as diabetes and cardiovascular disease (Vilariño‐García et al. 2024). More broadly, leptin is recognized to have a central metabolic role, with wide‐ranging effects on physiological systems (Perakakis et al. 2021).

Leptin research has been restricted by reliance on invasive venipuncture sampling, limiting application in low‐resource and remote settings and among vulnerable groups like children. Dried blood spot (DBS) sampling offers an alternative, minimally invasive approach for investigating circulating biomarkers (McDade et al. 2007). DBS samples offer advantages in stability, cost, and safety, proving valuable in human biology and related fields for feasibility in field settings and reduced participant burden (Urlacher et al. 2022). While a DBS‐based assay for measuring leptin was previously validated (Miller et al. 2006), its required components quickly became unavailable.

To advance leptin research, this study validates a widely available commercial ELISA kit measuring leptin in DBS.

## 2 Methods

This study was performed in the Human Evolutionary Biology and Health Lab at Baylor University, with human subjects' approval obtained from the Baylor University IRB.

### 2.1 Participants and Matched Sample Collection

Matched serum and finger‐prick DBS samples were obtained from a convenience sample of 40 adults (19–59 years old, 45% female) residing in McLennan County, Texas in 2020 as part of the Waco COVID Survey (Muehlenbein et al. 2023). Serum was collected via venipuncture using BD vacutainer tubes and centrifugation at 1000 g for 10 min. DBS samples were collected following standard procedures (McDade et al. 2007) on filter paper cards (Whatman #903, GE Healthcare). Cards were dried, sealed in airtight plastic bags with desiccant, and stored at −80°C until analysis.

### 2.2 Validation Procedures

#### 2.2.1 ELISA Kit Selection

The Human Leptin Quantikine QuicKit ELISA (R&D Systems, QK398, lot #P415277), designed for measuring human leptin in cell culture supernates, serum, and plasma was selected for DBS validation due to its wide availability, proven performance, and indicated suitability for DBS measurement. Important features include a reported sensitivity of 1.03 pg/mL and minimal sample volume requirement (50 μL per well).

#### 2.2.2 Leptin Measurement

Following preliminary testing to optimize leptin yield in DBS, two 3.2 mm^2^ punches per DBS sample were eluted overnight at 4°C in 150 μL of Calibrator Diluent in borosilicate glass test tubes. The following morning, eluted DBS samples were brought to room temperature, vortexed, and plated in duplicate alongside serum samples (diluted 1:100 with Calibrator Diluent). The kit protocol was then followed exactly.

#### 2.2.3 Serum/DBS Comparisons

To evaluate the relationship between leptin_DBS_ and leptin_serum_, matched samples were measured simultaneously, side‐by‐side on the same plate. Results were analyzed using Passing–Bablok regression (Passing and Bablok 1983). Bias was evaluated with Bland–Altman analysis (Bland and Altman 1986) by plotting the difference between leptin_serum_ and serum‐equivalent leptin_DBS_ (adjusted using the Passing–Bablok regression formula and correcting for dilution) against their average.

#### 2.2.4 Precision and Limit of Detection

Inter‐assay coefficient of variation (CV) was calculated using an in‐house DBS control with a leptin concentration of 73 pg/mL that was measured on each plate. The control was obtained using the same collection methods as the matched DBS samples. Intra‐assay CV was calculated using the average CV for all 40 DBS sample duplicates included in the study. The minimum detectable concentration of leptin was calculated as the average optical density of DBS blanks across all plates plus two standard deviations.

#### 2.2.5 Spike and Recovery

DBS samples from two individuals were spiked with low (+233 pg/mL) and high (+400 pg/mL) levels of leptin (dilutions of the kit standard). Neat samples from the same individuals served as references.

#### 2.2.6 Linearity of Dilution

Two DBS samples with relatively high concentrations of leptin were diluted at 1:2, 1:4, and 1:8 in Calibrator Diluent following the standard elution.

#### 2.2.7 Analyte Stability

Three DBS samples from different individuals each underwent 2, 4, and 8 freeze–thaw cycles, alternating between room temperature (26.2°C) for 8 h and −80°C for 16 h. Two DBS samples were each stored under hot (37°C) and room temperature (26.2°C) conditions for 72 h. A single measure was taken for each sample from both storage conditions at the end of the 72 h. Untreated samples were included as references, with all samples analyzed on a single plate for comparisons.

## 3 Results

### 3.1 Leptin Measurement and Matched Sample Comparison

**FIGURE 1:**
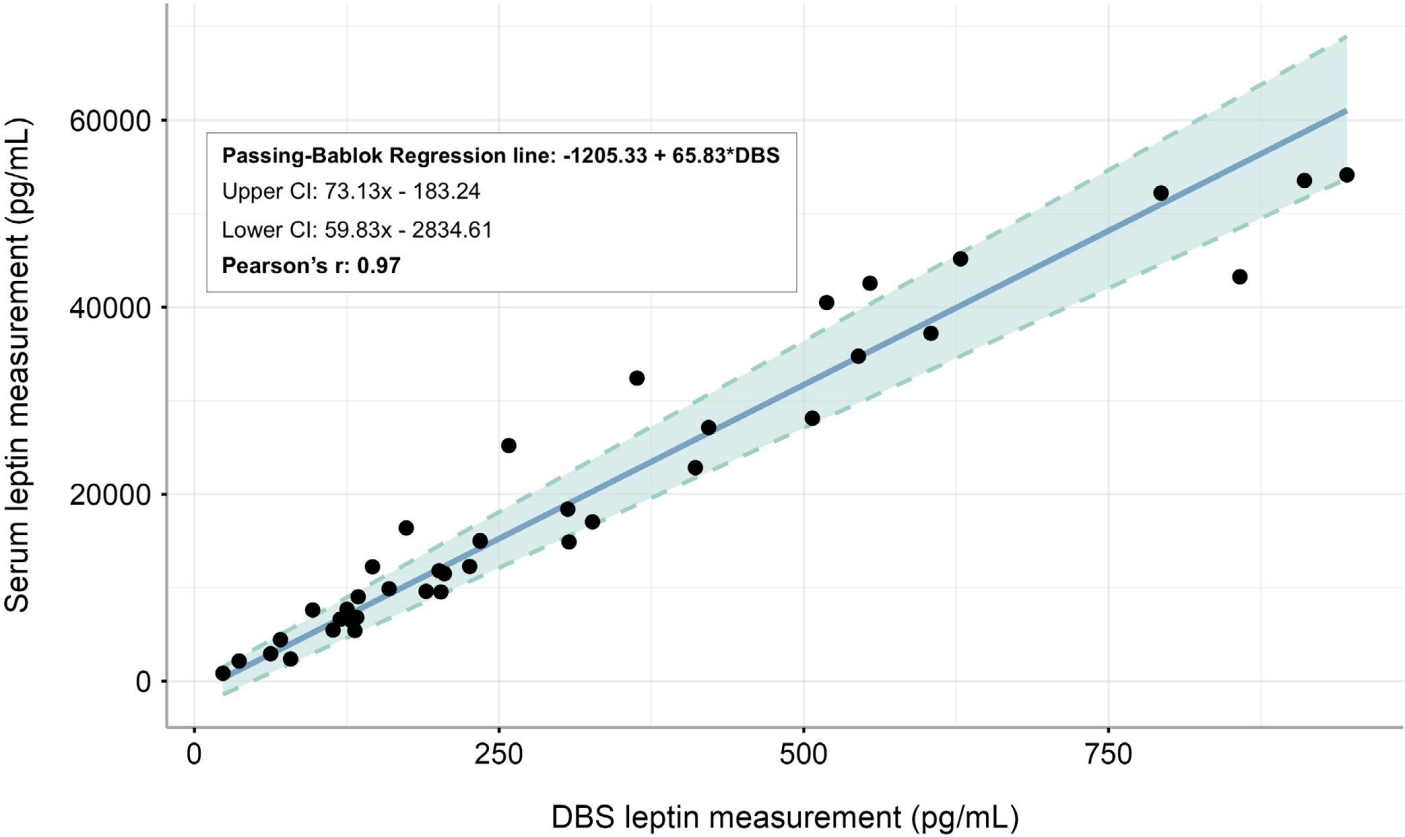
Passing–Bablok Regression for leptin measurement in matched DBS and serum samples (*n* = 40). Results demonstrate strong agreement between leptin measurements in DBS and serum.

**FIGURE 2:**
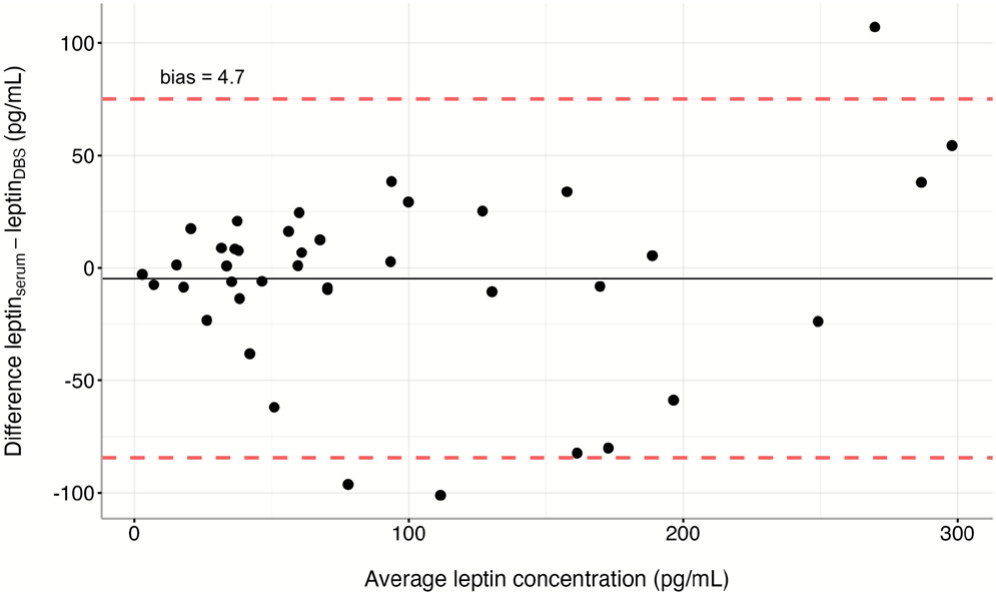
Bland Altman plot of the difference in serum leptin and DBS leptin (serum‐equivalent) values vs. the average of these values. Results demonstrate general agreement between both measures, with no apparent bias.

All DBS sample measurements fell within the range of the assay, with an average leptin_DBS_ of 312 pg/mL. Matched leptin_serum_ measures, correcting for dilution, averaged 19,518 pg/mL. Astrong linear relationship was observed between leptin_DBS_ and leptin_serum_ (Pearson's *R* = 0.97; Figure 1). Serum‐equivalent leptin_DBS_ had an average of 18 987 pg/mL. There was no obvious pattern of bias for the serum‐equivalent leptin_DBS_ vs. leptin_serum_ comparisons (bias = 4.7; Figure 2), indicating no systematic differences between methods.

### 3.2 Precision and Limit of Detection

Intra‐ and inter‐assay CVs were calculated as 3.3% and 2.0%, respectively. The assay lower limit of detection was 14.7 pg/mL.

### 3.3 Spike and Recovery

Leptin recovery after spiking ranged from 97.8% to 105.8% (average 102.3% ± 4.1%), indicating minimal interfering factors in the DBS matrix.

### 3.4 Linearity of Dilution

Leptin recovery for diluted samples ranged from 96.4% to 107.3% (average = 103.2% ± 4.2%), demonstrating good assay linearity.

### 3.5 Analyte Stability

Leptin recovery averaged 95.8% ± 3.0%, 93.3% ± 9.7%, and 95.0% ± 7.3% for 2, 4, and 8 freeze–thaw cycles, respectively, with no significant effects detected (all *p* > 0.05; Tukey HSD Test). Similarly, samples stored at room temperature (26.2°C) and hot (37°C) conditions for up to 72 h showed average recoveries of 94.4% ± 7.2% and 86.4% ± 7.9%, respectively, with no significant differences from controls (all *p* > 0.05; Tukey HSD Test).

## 4 Discussion

This study demonstrates that a widely available commercial ELISA kit can reliably measure leptin in minimally invasive finger‐prick DBS. Leptin was successfully measured in all DBS samples from a range of adults (19–69 years, 45% female), indicating the assay's broad utility. Matched leptin_serum_ and leptin_DBS_ showed strong agreement (*R* = 0.97) with minimal bias, similar to a prior leptin DBS assay validation using now‐unavailable components (*R* = 0.98; Miller et al. 2006). Additional validation testing indicated strong precision, linearity, and minimal DBS matrix interference.

Leptin in DBS samples demonstrates good stability. No significant changes in leptin_DBS_ were detected over eight freeze–thaw cycles and up to 72 h at both room temperature and hot conditions. This finding contrasts with Miller et al. (2006), who reported degradation in leptin_DBS_ after 24 h in hot conditions, perhaps owing to differences in assay sensitivity. However, it is important to note that Miller et al. assessed stability using a threshold as its criteria (10% below baseline) rather than statistical significance. Applying the same criteria to the present validation, one sample exceeded the stability threshold after 72 h in hot conditions. Future studies should more sensitively evaluate the stability of leptin in hot conditions.

Reliable measurement of leptin in DBS samples enables leptin research with populations and groups for whom traditional, invasive venipuncture sampling is impractical. This includes children and other vulnerable/marginalized groups (e.g., low‐resource settings, Indigenous populations, etc.). While leptin's role in appetite regulation, energy balance, and chronic disease risk in industrialized adult populations is well documented (Perakakis et al. 2021), reliance on invasive venipuncture has hindered leptin research across the wider range of human environmental and lifestyle diversity. The limited existing evidence suggests that leptin's energetic role may vary across populations (Kuzawa et al. 2007; Moore et al. 2002), pointing to potential flexibility and adaptive variation in leptin signaling (Bribiescas 2005; Sharrock et al. 2008).

## 5 Conclusions

Leptin can be reliably and stably measured in DBS samples using a modified protocol with a commercially available ELISA kit. Future research can apply the DBS‐based approach for leptin measurement presented here to deepen insight into this hormone's role in shaping human phenotype and health.

## Funding

This work was supported by the CIFAR Azrieli Global Scholars Program, Cooper Foundation of Waco, Bernard and Aubre Rapoport Foundation of Waco, and Waco Family Medicine.

## Conflicts of Interest

The authors declare no conflicts of interest.

## Data Availability

The data that support the findings of this study are available from the corresponding author upon reasonable request.
